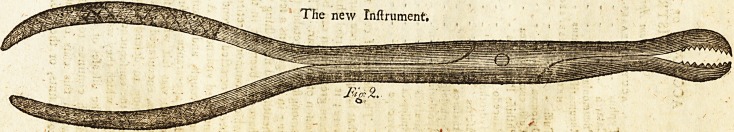# To Dr. Bradley

**Published:** 1802-06

**Authors:** T. Chevalier

**Affiliations:** South Audley Street


### To Dr. BRADLEY.

DEAR SIR,

HAVING been frequently diſappointed in attempts to extract balls, and exfoliated pieces of bone, by the forceps in common uſe, I was induced to attempt an improvement in the conſtruction of theſe inſtruments, and requeſted Mr. Stodart to make ſeveral pairs on a different plan, which you will find repreſented in the drawing I have encloſed, and which has been found perfectly to anſwer its intention.

The old ball forceps (ſee fig. 1.) are faulty in ſeveral reſpects. 1ſt. The principal length of the inſtrument is thrown into the blade inſtead of the handle, which is in conſequence too ſhort, and renders the gripe weak and uncertain. 2. The two parts of the handle touch each other when the inſtrument is ſhut; and very much limits its power. 3. The rings of the handle cramp the fingers, to which only they are adapted, for they make it impoſſible to hold the inſtrument firmly in the palm of the hand. From theſe cauſes, if a ball, or other extraneous ſubſtance, be faſt driven into any part, the forceps perpetually ſlip from it, and it becomes extremely difficult to diſlodge and bring it away.

The new forceps, (ſee fig. 2.) remedy all theſe inconveniences. 1. Their principal length is thrown into the handle, which adds very much to the power of the inſtrument, and conſequently to the firmneſs with which it will hold any ſubſtance which it is neceſſary to extract. 2. The two parts of the handle are divergent, ſomewhat broad and roughened, and made without rings, ſo that they can be held by the whole hand, and uſed with a much greater purchaſe. By this mode of conſtructing the inſtrument more metal alſo can be employed, ſo as to render it more ſolid, capable of being harder tempered, and thus of courſe, much ſtronger than the other, without being inconvenient from its bulk.

The army inſtrument caſes have lately been furniſhed with ſtrait and curved forceps of this conſtruction, by order of the Surgeon General. Forceps on the ſame plan are alſo now made by Mr. Stodart and Mr. Savigny for the extraction of exfoliated bones, polypi, of the ſtone in the operation of Lithotomy, and for other purpoſes; and I flatter myſelf it will be found a conſiderable improvement in this claſs of inſtruments.

*South Audley Street,* I am. &c.

## Figures and Tables

**Fig. 1. f1:**
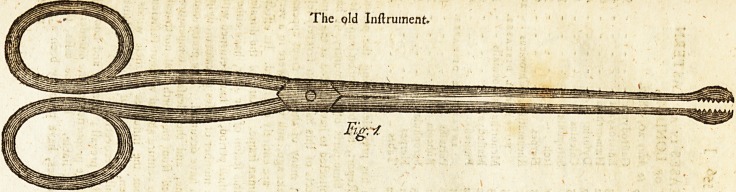


**Fig. 2. f2:**